# Controlling the Self-Assembly and Material Properties of β-Sheet Peptide Hydrogels by Modulating Intermolecular Interactions

**DOI:** 10.3390/gels9060441

**Published:** 2023-05-26

**Authors:** James P. Warren, Matthew P. Culbert, Danielle E. Miles, Steven Maude, Ruth K. Wilcox, Paul A. Beales

**Affiliations:** 1 School of Chemistry, University of Leeds, Leeds LS2 9JT, UK; j.p.warren@leeds.ac.uk (J.P.W.); cm14mpc@leeds.ac.uk (M.P.C.); danielle.miles@shu.ac.uk (D.E.M.); steven.maude@phc.ox.ac.uk (S.M.); 2 School of Mechanical Engineering, University of Leeds, Leeds LS2 9JT, UK; 3 Institute of Medical and Biological Engineering, University of Leeds, Leeds LS2 9JT, UK; 4 Bragg Centre for Materials Research, University of Leeds, Leeds LS2 9JT, UK; 5 Astbury Centre for Structural Biology, University of Leeds, Leeds LS2 9JT, UK

**Keywords:** β-sheet, self-assembly, peptides, hydrogels, rheology

## Abstract

Self-assembling peptides are a promising biomaterial with potential applications in medical devices and drug delivery. In the right combination of conditions, self-assembling peptides can form self-supporting hydrogels. Here, we describe how balancing attractive and repulsive intermolecular forces is critical for successful hydrogel formation. Electrostatic repulsion is tuned by altering the peptide’s net charge, and intermolecular attractions are controlled through the degree of hydrogen bonding between specific amino acid residues. We find that an overall net peptide charge of +/−2 is optimal to facilitate the assembly of self-supporting hydrogels. If the net peptide charge is too low then dense aggregates form, while a high molecular charge inhibits the formation of larger structures. At a constant charge, altering the terminal amino acids from glutamine to serine decreases the degree of hydrogen bonding within the assembling network. This tunes the viscoelastic properties of the gel, reducing the elastic modulus by two to three orders of magnitude. Finally, hydrogels could be formed from glutamine-rich, highly charged peptides by mixing the peptides in combinations with a resultant net charge of +/−2. These results illustrate how understanding and controlling self-assembly mechanisms through modulating intermolecular interactions can be exploited to derive a range of structures with tuneable properties.

## 1. Introduction

Hydrogels are a three-dimensional network of polymer chains swollen with water [1,2,3]. Self-assembling peptides can be used to form versatile and tuneable hydrogels with an intrinsic biocompatibility and a range of biomechanical and biophysical properties [4,5,6,7]. This makes them suitable for use in a range of applications such as early-stage interventions for musculoskeletal disorders, drug delivery platforms or scaffolds for cell therapies [8,9]. Some practical applications where hydrogels have been used are in the early-stage repair of degenerated soft tissues such as cartilage and intervertebral disc [4,10]. In order to fully utilise these biomaterials for practical applications, the design principles that drive self-assembly and determine their material properties need to be better understood.

A number of design variables that affect self-assembly and material properties of different families of peptides have been investigated previously. For example, for alpha-peptides, it has been shown that tuning hydrophobic and π-π interactions through the amino acid sequence was critical to determining whether a percolating gel phase could form [11]. Changing the amino acid sequence has also been found to change the secondary structures formed, as the peptides self-assemble in the case of a short eight-residue peptide, GV8, which causes transitions between α-helical and different β-sheet structures [12], and results in differences in the rheological properties. For the RADA family of peptides that self-assemble due to alternating hydrophobic and negatively charged peptides, the effect of adding proline amino acids at both the C- and N- terminals was shown to increase the peptide solubility, sol–gel transition ability, and the storage modulus through the effect of proline–proline-type self-assembly. These examples illustrate how minor changes to the peptide structure can influence the resulting properties on different scales [13].

**Figure 1:**
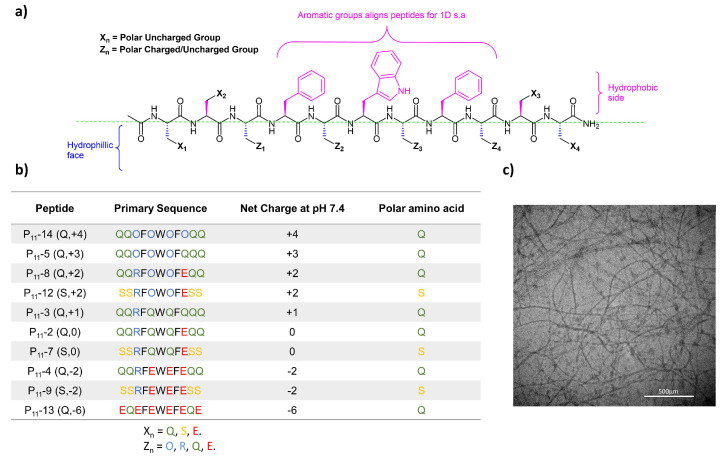
Peptide structures in the P_11_ series. (**a**) Generic design principles for P_11_ β-sheet forming peptide structures. (**b**) Table of peptides investigated in this study with structures and charges at physiological pH. (**c**) TEM image of P_11_-12 in 130 mM saline (~6 mM) at pH 7.4 showing a self-assembled fibrous hydrogel network. Scale bar is 500 µm.

Here, we investigate the family of P_11_ peptides that have been developed to self-assemble spontaneously to form β-sheet 3D hierarchical structures. Figure 1 shows the generic structure of this class of peptides, as well as the amino acid sequences, and net charge of different P_11_ peptides [14].

The P_11_ peptides are designed with a hydrophobic aromatic centre on one side and a hydrophilic side to promote the formation of anti-parallel β-sheets, and then, through a hierarchical self-assembly mechanism, form a fibrous multi-micron scale network. An example TEM micrograph of a P_11_ peptide hydrogel (P_11_-12) illustrates the fibrous network that forms during self-assembly (Figure 1c). Previous work has highlighted that charge is an important factor when assessing the biocompatibility of positively charged self-assembling hydrogels, including P_11_-8 [15].

This work builds on existing knowledge by focusing on self-assembly under physiological buffer conditions (pH, ionic strength). We investigate the effects of altering two different design variables on the relationship between molecular scale and macroscale properties: (1) the overall peptide charge; (2) the hydrogen bonding interactions, achieved through changing the terminal amino acids from glutamine (Q) to serine (S). The former is a mechanism to tune repulsive interactions between peptides and the latter modulates attractive intermolecular interactions. We demonstrate that controlling the balance between these attractive and repulsive interactions is critical in controlling the self-assembly of P_11_ peptides and determining conditions under which peptide hydrogels can form.

## 2. Results and Discussion

The results are presented to, firstly, show the effect of charge on intermolecular repulsion under physiological self-assembly of glutamine-containing peptides, then, the effect of molecular attraction is explored through the degree of hydrogen bonding by comparing three different serine-containing peptides to three glutamine-containing equivalent peptides. Finally, based on these findings, we demonstrate the formation of a hybrid hydrogel formed from a mixture of non-gelling P_11_ peptides. To aid the reader in correlating the peptide number (*N*) in the P_11_ series with its charge, peripheral amino acid type, and net charge, we use the nomenclature P_11_-*N* (*a*,*b*), where *a* are the peripheral amino acids (Q for glutamine; S for serine) and *b* is the net charge of the peptide at the physiological pH.

### 2.1. Control of Intermolecular Repulsion Is Critical for the Self-Assembly of Peptide Hydrogels

**Figure 2:**
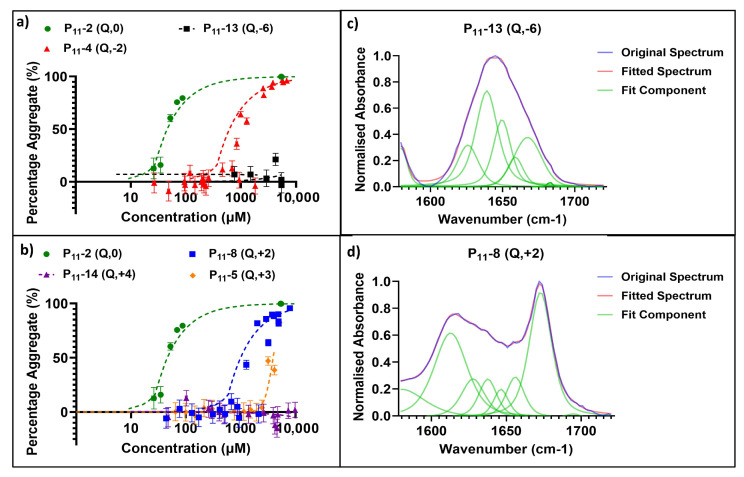
Characterisation of glutamine-containing P_11_ peptide self-assembly. (**a**,**b**) Self-assembly curves from ^1^H NMR analysis. Self-assembly at a range of P_11_ peptide concentrations and overall net charge are shown in order to determine c* of negative- (**a**) and positive- (**b**) charged peptide systems. (**c**) Secondary structure analysis of the Amide I region in FT-IR spectra, where the various components are illustrated for a peptide that does not self-assemble: P_11_-13 (Q,−6), [P_11_-13] ~5 mM. (**d**) Secondary structure analysis of the Amide I region in FT-IR spectra, where the various components are illustrated in a self-assembling peptide system: P_11_-8 (Q,+2), [P_11_-8] ~5 mM.

Our series of glutamine-based peptides had overall charges (at pH 7.4) from −6 to +4 (Figure 1). Each peptide varies in critical aggregation concentration (c*) as shown by the self-assembly curves derived from the ^1^H NMR experiments (Figure 2). The c* for each peptide is the minimum peptide concentration required for peptide self-assembly, where favourable intermolecular interactions between peptides overcome the entropy of mixing, which favours dispersion of the peptides in the monomer state. When above this concentration, self-assembled aggregates will coexist in dynamic equilibrium with a background critical concentration of peptide monomers in solution. The method of calculating c* for each peptide is demonstrated in an example in Section 4.5.3. The neutrally charged P_11_-2 (Q,0) peptide possessed the lowest c* (14 ± 3.2 μM), while the two peptides with the extreme charges, P_11_-13 (Q,−6) and P_11_-14 (Q,+4), showed no self-assembly within the concentration range investigated (~1 μM–100 mM). The peptides with +/− 2 overall charge, P_11_-4 (Q,−2) and P_11_-8 (Q,+2), possessed similar c*: 310 ± 72 and 400 ± 100 μM, respectively [14].

The secondary structures of these peptides under conditions of self-assembly, where applicable, were characterised by FT-IR spectroscopy. Figure 2 shows example spectra for a peptide that does not self-assemble (P_11_-13 (Q,−6); Figure 2c) and a peptide that has undergone self-assembly (P_11_-8 (Q,+2); Figure 2d). In the case of the extremely charged peptide systems (−6/+4), the peaks in the Amide I region typically associated with β-sheet formation, 1613–1630 cm^−1^ and 1682–1690 cm^−1^, were not present in the FT-IR spectra. Instead, the spectrum is predominantly random coil structure. For peptides that do self-assemble (based on our NMR data), the IR spectra show characteristic peaks for β-sheet content that increase in area as the proportion of β-sheet in the system increases.

**Figure 3:**
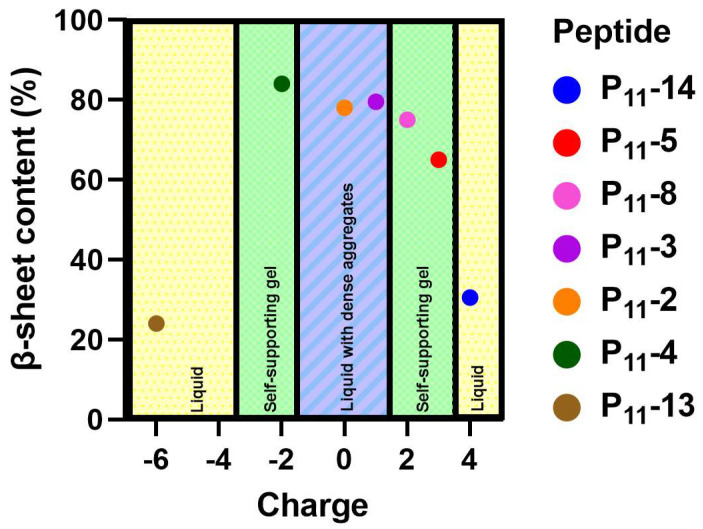
A phase diagram showing the relative β-sheet content versus overall charge of each peptide system. The physical state of each of these systems is categorised into whether a gel or liquid formed.

The calculated β-sheet content for each glutamine-containing peptide along with the macroscopic physical state of the sample are shown in the phase diagram in Figure 3. Here, we see that the neutral P_11_-2 (Q,0), and +/− 2 charged P_11_-4 (Q,−2), and P_11_-8 (Q,+2) form similar β-sheet compositions, ranging from 75–85%. The negatively charged P_11_-4 (Q,−2) showed the highest β-sheet proportion at 85%, while the positively charged P_11_-8 (Q,+2) showed the lowest proportion at 75%. The neutral P_11_-2 (Q,0) was intermediate between these values at 78% β-sheet content. The charged peptides, P_11_-4 (Q,−2) and P_11_-8 (Q,+2), both form a self-supporting gel. However, despite the similar β-sheet content, P_11_-2 (Q,0) is observed to form a free-flowing fluid with visible flocculates present. Therefore, β-sheet assembly of P_11_ peptides is not a sufficient criterion for formations of a percolating gel structure. We interpret that long-range electrostatic repulsion in the self-assembled filaments is required for a self-supporting gel network to form throughout the sample. Without this repulsive contribution to the system, dense flocculates preferentially form.

### 2.2. Molecular Attraction Can Modulate Gel Self-Assembly and Mechanics

Having established that controlled long-range intermolecular repulsion is important for the gelation of P_11_ peptides, we wished to improve our understanding of how the strength of intermolecular attractions, essential to drive the self-assembly process, further modulate P_11_ hydrogel properties. To this end, the role of uncharged polar amino acids in symmetric positions *X*_1_, *X*_2_, *X*_3_, and *X*_4_ in the peptide sequence (see Figure 1) were explored. Specifically, the hydrogen bonding interactions between peptides were modulated by swapping the strongly hydrogen-bonding glutamine side-chain for the weaker hydrogen-bonding side-chain of serine. This difference in hydrogen bonding interactions of polar amino acid residues is well-established and known as the “polar zipper” effect for glutamines [16]. Three serine-based peptides were designed and characterised: P_11_-7 (S,0), P_11_-9 (S,−2), and P_11_-12 (S,+2). These peptides are comparable to our three glutamine-based peptides: P_11_-2 (Q,0), P_11_-4 (Q,−2), and P_11_-8 (Q,+2).

**Figure 4:**
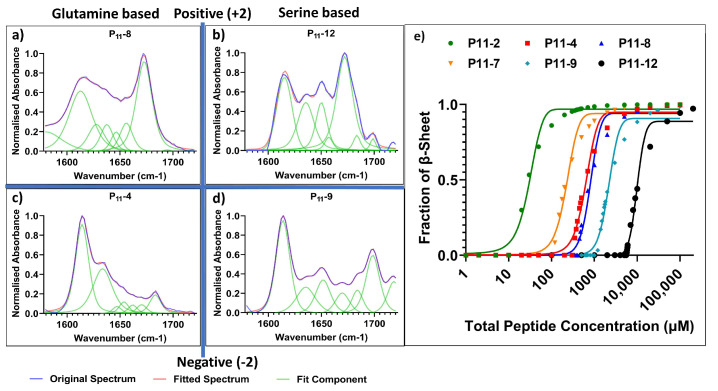
Comparison of the self-assembly of glutamine- and serine-containing P_11_ peptides. (**a**–**d**) FT-IR spectra (concentration ~5 mM of each peptide) with secondary structure analysis of Amide I region for: (**a**) glutamine-based P_11_-8, (**b**) serine-based P_11_-12, (**c**) glutamine-based P_11_-4 and (**d**) serine-based P_11_-9, (**e**) ^1^H NMR analysis of self-assembly and determination of c*.

In the case of the two neutrally charged peptides, P_11_-2 (Q,0) and P_11_-7 (S,0), the lowest c* values were observed (14 μM and 110 μM, respectively) (Figure 4). Notably, the reduction in attractive hydrogen bonding interactions between serine residues increases the c* for self-assembly. Despite the low c* values, neither system formed self-supporting hydrogels. This is consistent with our interpretation that long-range charge repulsion is required for the gelation of these peptides.

For self-supporting peptide hydrogels with an overall net charge of magnitude 2 (independent of the sign of the charge), glutamine-based peptides had the lowest c*. When decreasing the extent of attractive hydrogen bonding interactions from glutamine- to serine-based peptides, the c* increased. This is made clear from the c* for P_11_-12 (S,+2) of 2300 µM compared to 400 µM for P_11_-8 (Q,+2), and the c* for P_11_-9 (S,−2) of 1160 µM compared to 310 µM for P_11_-4 (Q,−2) (Figure 4). Only small non-significant differences in average β-sheet content were observed between glutamine- and serine-based peptides, P_11_-4 (84 ± 5%), P_11_-8 (75 ± 4%), P_11_-12 (74 ± 10%), and P_11_-9 (75 ± 10%).

Despite weaker hydrogen bonding attraction between peptides, the serine-based peptides still form self-supporting hydrogels at concentrations above c*. This implies that an overall charge of magnitude 2 is the more dominant factor in determining whether a self-supporting, percolating gel network is formed [17]. These observations and conclusions reinforce predictions from a previously reported theoretical model that attempted to determine the self-assembly energetics of this system. This model predicted that the overall charge of the system had a greater influence over the degree and strength of self-assembly rather than the precise amino acid choice [18].

**Figure 5:**
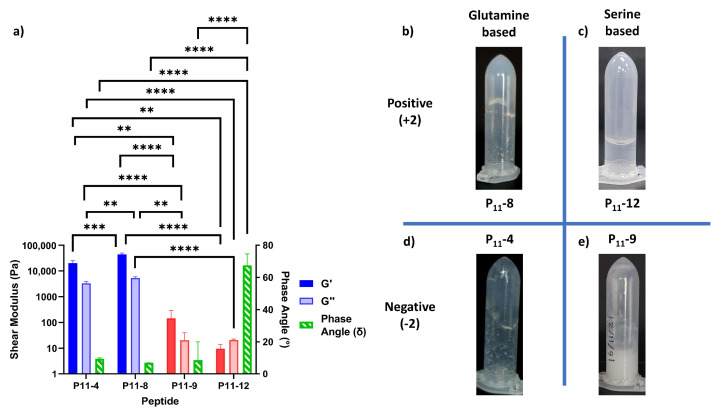
Rheological characterisation of P_11_ hydrogels. (**a**) The shear moduli (elastic component, *G*′, and viscous component, *G*″) and the phase angles (green) for peptides with a ±2 charge and polar uncharged amino acids glutamine (blue) or serine (red). Statistical significance is tested using a one-way ANOVA with a post hoc Tukey comparison (** *p* < 0.01; *** *p* < 0.005; **** *p* < 0.001). (**b**–**e**) Tube inversion studies of peptide hydrogels, qualitatively testing their resistance to flow under gravity, (**b**) P_11_-8, (**c**) P_11_-12, (**d**) P_11_-4, and (**e**) P_11_-9.

Even though the change in polar amino acid does not affect hydrogel formation, these chemical changes in peptide structure can be used to tune the physical properties of the hydrogel. In particular, the mechanical properties of the hydrogel are dependent on the cohesive energy between peptide molecules. The rheological properties of these P_11_ peptide hydrogels are measured and compared in Figure 5. The elastic moduli (*G*′) for the glutamine-based peptides (P_11_-4 (Q,−2) and P_11_-8 (Q,+2)) are higher than those of the comparative serine-based peptides (P_11_-9 (S,−2) and P_11_-12 (S,+2)) by two or three orders of magnitude, respectively. From simple tube inversion experiments, it can also be seen that the serine-based hydrogels are weak enough to break and flow under gravity (also demonstrated by the high phase angle of P_11_-12 (S,+2) gels). This is comparable with glutamine-based peptide gels that do not flow under gravity. Taken together, this demonstrates that the increased intermolecular hydrogen bonding in a glutamine-based peptide generates a stiffer gel than a corresponding serine-based peptide gel under the same conditions.

### 2.3. Highly Charged Peptides can Form Gels When Mixed to Form Composites of Lower Net Charge

While highly charged peptides were not observed to self-assemble under physiological conditions, our finding that the overall magnitude of the charge is the dominant factor for gelation suggested that these peptides might gel in a combination where the overall net charge of the peptide assemblies was reduced to within the range required for gel formation (~+/−2). To test this hypothesis, we combined the peptides P_11_-13 (Q,−6) and P_11_-14 (Q,+4) in an equimolar ratio to see if this could induce self-assembly. Neither peptide underwent self-assembly in isolation under physiological conditions at any concentration.

**Figure 6:**
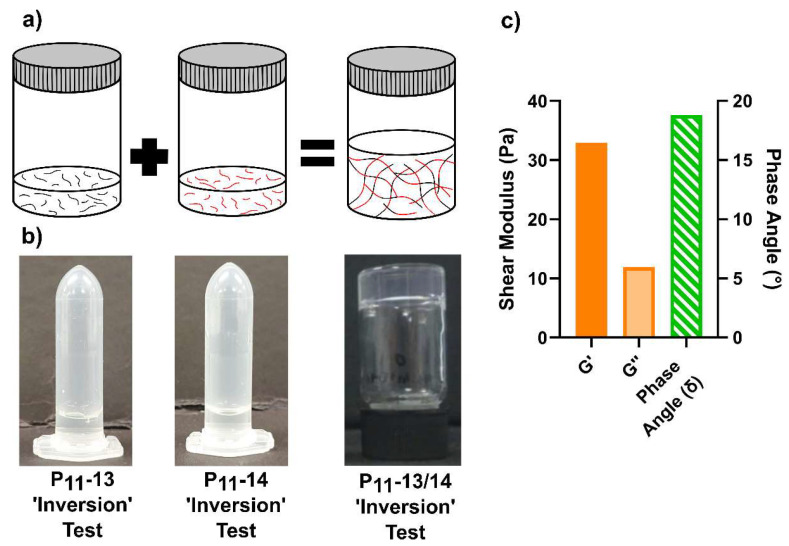
Formation and characterisation of hybrid P_11_ peptide hydrogels. (**a**) Schematic representation with photographic examples of P_11_-13 (Q,−6) and P_11_-14 (Q,+4) as single component and combined systems. (**b**) The formation of a gel post-mixing is evident through the tube inversion test (photographs). (**c**) Rheological characterisation further demonstrates the formation of a gel in the hybrid mixture of P_11_-13 (Q,−6) and P_11_-14 (Q,+4) peptides.

On combining P_11_-13 (Q,−6) and P_11_-14 (Q,+4), the composite system now has an average net charge of −2 per peptide, within the range where hydrogels are formed in single-component peptide systems, c.f. P_11_-4 (Q,−2). Indeed, this combination of highly charged peptides led to the formation of a self-supporting hydrogel as shown in Figure 6. The G′ and G″ of the P_11_-13/14 (Q,−2) hydrogel was considerably lower than that of P_11_-4 (Q,−2) by three orders of magnitude. The *G*′ of the composite P_11_-13/P_11_-14 (Q,−2) system was ~32 Pa and *G*″ ~12 Pa, compared to P_11_-4 (Q,−2) where *G*′ and *G*″ were 12 and 4 kPa, respectively. The phase angles of both systems were not dissimilar, P_11_-13/14 (Q,−2) ~18° while P_11_-4 (Q,−2) is ~15°. However, while the moduli of the composite system were greatly reduced, likely due to the high charge density in the hybrid hydrogel, a self-supporting hydrogel that could resist flow under gravity formed upon combining these non-gelling individual peptides.

## 3. Conclusions

In the design of self-assembling peptide systems, changes to the amino acid sequence alter the non-covalent interactions that drive self-assembly [19]. For P_11_ peptides, two fundamentally important interactions in their self-assembly into β-fibril hydrogels are the hydrogen bonding between the peptide backbones and the hydrophobic interactions between the side-chains of core amino acids in the sequence [20]. These attractive intermolecular interactions oppose the resisting influence from the entropy of the mixing of the system, which favours disassembly [21].

However, additional non-covalent interactions can be controlled between these peptides to further modulate their self-assembly and tune the properties of the composite material. Here, we set out to investigate the roles of long-range electrostatic repulsion and short-range attractive interactions through additional hydrogen bonding interactions in the amino acid side groups [22]. The overall charge of the peptide sequence was altered from neutral to extremes (+4 and −6) through the removal or addition of polar amino acids (glutamic acid, ornithine, and arginine), while amino acid side-chain hydrogen bonding was modulated at the four peripheral amino acid positions through the uncharged polar amino acids serine and glutamine [23].

The overall magnitude of the peptide charge, independent of its sign, is the dominant factor in driving self-assembly and hydrogel formation [24]. As the net charge of the peptide decreases, the critical concentration for self-assembly (c*) also decreases with the lowest c* being measured in the neutral P_11_-2 (Q,0) and P_11_-7 (S,0) systems.

**Figure 7:**
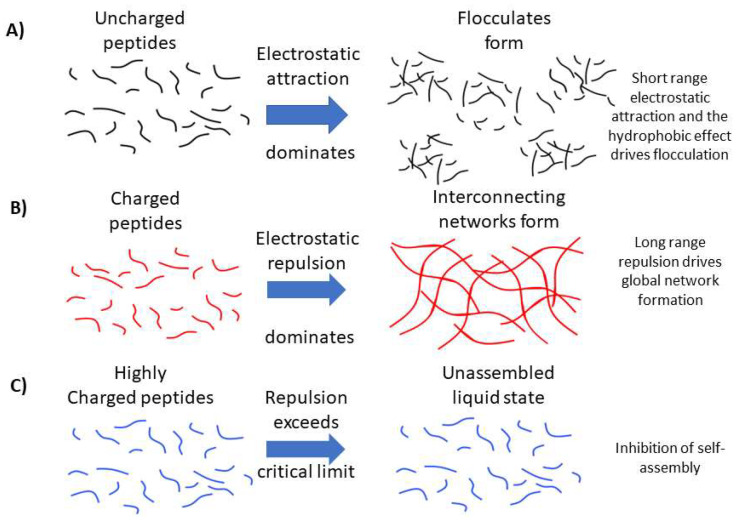
Schematic representation of our hypothesis for the (**A**) role of long-range electrostatic repulsion between peptide fibrils in the formation of open, extended hydrogel networks. (**B**) Conversely, we propose that short-range attractions dominate between assembled uncharged peptide filaments, leading to dense flocculates that are unable to percolate the solution and form a space-filling gel network. (**C**) In the case of highly charged peptides, we propose that electrostatic repulsion exceeds a critical limit, resulting in inhibition of self-assembly.

Our work makes it clear that a low c* is not an indicator that a peptide system will form a self-supporting hydrogel network. In order to form a self-supporting hydrogel network, in this P_11_ class of peptides, an overall magnitude of molecular charge of ~2 is required, which we hypothesise is due to the requirement of long-range repulsion between β-fibrils favouring a well-hydrated open network of a self-supporting gel, as represented in Figure 7 [21,22]. Indeed, this conclusion is reinforced by the discovery that mixtures of oppositely charged peptides with a net average charge of magnitude 2 could also self-assemble into hydrogels, even though these peptides do not individually self-assemble or form gels. When comparing between peptide systems with a +2 or −2 charge, those with glutamine as the polar amino acid resulted in a lower c* and larger gel stiffness (*G*′ and *G*″) due to the additional hydrogen bonding interactions enhancing the cohesive energy of the structures [25]. Similarly, the increased cohesive energy in the self-assembly of glutamine-rich peptides decreases the c* for self-assembly, compared to serine-rich analogues [26].

This work demonstrates strategies for the control of the self-assembly and material properties of P_11_ peptide hydrogels, which will allow engineering of optimal hydrogel formulations for a range of applications from cell culture scaffolds to regenerative medicine [27,28]. The ability to control the self-assembly and material properties of P_11_ peptide hydrogels also enables their use as injectable biomaterials with in situ self-assembly for clinical applications to augment or repair degenerated soft tissues [29].

## 4. Materials and Methods

### 4.1. Materials

All the peptides were either supplied through CPC Scientific (Sunnyvale, CA, USA) (P_11_-2, P_11_-3, P_11_-5, and P_11_-7), Polypeptide Laboratories (Strasbourg, France) (P_11_-13 and P_11_-14), or CS Bio (Menlo Park, CA, USA) (P_11_-4, P_11_-8, P_11_-9 and P_11_-12). Sodium chloride, HCl, NaOH, D_2_O, DCl, and NaOD were purchased from Sigma Aldrich (Gillingham, UK). Phosphate buffered saline (PBS) was purchased from Oxoid, Thermofisher Scientific (Waltham, MA, USA).

### 4.2. Weighing

A Mettler AE240 balance was used to measure masses greater than ≈1 mg, while masses less than ≈1 mg were weighed on a Sartorius SC2 balance.

### 4.3. Dissolution

Aqueous solvent was added to weighed peptide. Unless otherwise specified, the solvent contained 130 mM NaCl (Fisher Scientific, Waltham, MA, USA) dissolved in pure water (resistivity 18.2 MΩ cm during dispensation). After sealing sample vials with Parafilm^®^, they were vortexed for ≈30 s using a Scientific Industries Vortex Genie 2 vortex mixer, and placed in a Bandelin Sonorex RK52H sonicator for ≈30 min.

### 4.4. pH Measurement and Adjustment

Samples were adjusted to a pH or pD range 7.4 ± 0.3, unless otherwise specified. Sample pH was determined using either a WPA CD720 m and a CMW711 semi-micro single junction probe, or a Sartorius Docu-pH meter and a VWR symphony semi-micro combination double junction probe (part 14231-178). Microlitre aliquots of typically 0.1 or 1.0 M HCl or NaOH were added to solutions to alter their pH (for deuterated solutions, DCl or NaOD were used). After each addition of acid or base, the solution was briefly vortexed, and then its pH or pD rechecked [30].

### 4.5. Proton Nuclear Magnetic Resonance Spectroscopy Measurement

#### 4.5.1. Solution Preparation

Solutions were prepared as detailed in Section 4.3, but using 130 mM NaCl in D_2_O, and with an internal reference of either 0.125 or 1.25 mM 3-(trimethylsilyl) propionic acid-2,2,3,3-d4 (TMSP) (Cambridge Isotope Laboratories, Tewksbury, MA, USA).

#### 4.5.2. Equipment Setup and Data Acquisition

Samples were transferred to NMR tubes (Norell XR-55-7 5 mm borosilicate). Proton NMR spectra were acquired with a Bruker DPX 300 spectrometer(Billerica, MA, USA) controlled by XwinNMR software. A presaturation program (Bruker zgpr program) was used to minimise the water peak. A total of 1024 scans were recorded per spectrum.

#### 4.5.3. Processing of Data

**Figure 8:**
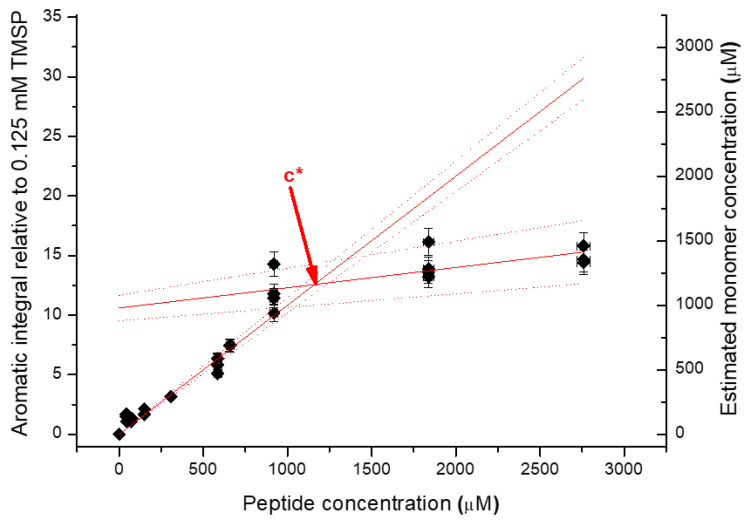
An example of the process of determining the c* value from NMR data.

Data were analysed with MestreNova version 5.3.3-5204 (MestreNova Research SL), running on the Ubuntu Linux distributions. The reference peak was assigned the chemical shift of 0.00 parts per million (ppm). Integral values represent the area of the aromatic region (6.9 to 7.8 ppm) relative to the TMSP reference peak; usually these integrals were linearly corrected automatically. In order to determine the critical concentration, the estimated monomer concentration was required. To determine the estimated monomer concentration, the integral of the aromatic peak was divided by the slope of peptide concentration vs. integral, for the monomer/linear regime. To determine the critical concentration, aromatic integral vs. monomer concentration as a function of peptide concentration was plotted. Two lines were fitted, one for monomers at low concentration and one for when aggregation at high concentration occurs. The crossover point of these two lines equates to the c* value. An example of this is shown in Figure 8.

The self-assembly curves were constructed by subtracting the estimated monomer concentration (derived from the aromatic integral) from the total peptide concentration to give the aggregate percentage.

### 4.6. Transmission Infra-Red Spectroscopy Measurement

#### 4.6.1. Solution Preparation

Solutions were prepared as in Section 4.3 in D_2_O, all containing NaCl, while some additionally contained NMR reference compounds.

#### 4.6.2. Equipment SETUP and Data Acquisition

A Thermo Nicolet 6700 FTIR spectrometer (Thermo Electron Corporation, Waltham, MA, USA), controlled with OMNIC 7.3 SP1 (Thermo Scientific) software, was used to record FTIR spectra. Liquid samples were held in a Thermo HT-32 demountable cell with CaF_2_ windows (Thermo Scientific, Waltham, MA, USA) and a copper or lead spacer between the windows (usually a 0.025 mm copper spacer). A total of 40 µL of liquid sample was injected into the cell. A background air spectrum, using an empty sample compartment (no cell), was recorded just before a sample was analysed. The spectra consist of 32 scans recorded at a resolution of 4 cm^−1^. Spectra were collected at room temperature, with the cell temperature measured with a Thermo Scientific CAL 9500 thermostat (Waltham, MA, USA).

#### 4.6.3. Processing of Spectra

Peptide spectra were processed with OMNIC 7.3 SP1 software (Thermo Scientific). A D_2_O spectrum recorded using the same path length spacer as was used for the sample, was subtracted from the sample spectrum. Subtracted spectra were then baseline corrected with a manually selected baseline, whose points were fixed to spectral values and interpolated using a spline function. To fit the amide I band (in the 1720–1580 cm^−1^ range), baseline corrected spectra were processed with the OMNIC Peak Analysis tool [31,32]. Gaussian–Lorentzian peak functions were fitted and optimised. Once converged, these peaks were recorded as the relative areas of each peak and associated to a specific secondary structure or amino acid residue [4,33,34].

### 4.7. Rheological Measurements

All the rheological measurements were performed on a Malvern Kinexus Pro rheometer (Malvern, Worcestershire, UK) with a cone–plate geometry (diameter 50 mm, cone angle 1°, and gap height of 0.033 mm) with ~700 μL sample required per test. Temperature was maintained at 25 °C and a solvent trap was used to minimise evaporation of the peptide samples. Loaded samples were left for 15 min to equilibrate before testing.

To measure the dynamic moduli of each hydrogel, the linear viscoelastic region (LVER) was first identified through amplitude sweeps over the shear strain range of 0.01 to 100% at frequencies of 1 Hz and 20 Hz, respectively. At these frequencies, shear strains ranging from 0.1–0.5% were found to consistently lie within the LVER for each specific peptide where the elastic modulus (*G*′) and viscous modulus (*G*′′) remained constant. These settings were away from the sensitivity limits of the instrument where noise is observed in the measurements. Artefacts were avoided by checking for no drops in the elastic modulus or peaks in the viscous modulus indicative of sample slippage.

The dynamic moduli of the hydrogels were then measured as a function of frequency between 1 and 20 Hz using the identified strain of 0.1%.

## Data Availability

The data that support the findings of this study are openly available in The White Rose Repository.
